# Prevalence and predictors of giving birth in health facilities in Bugesera District, Rwanda

**DOI:** 10.1186/1471-2458-12-1049

**Published:** 2012-12-05

**Authors:** Shahrzad Joharifard, Stephen Rulisa, Francine Niyonkuru, Andrew Weinhold, Felix Sayinzoga, Jeffrey Wilkinson, Jan Ostermann, Nathan M Thielman

**Affiliations:** 1 Department of Surgery, Division of General Surgery, University of British Columbia, Vancouver, BC, Canada; 2 Department of Clinical Research, Kigali University Teaching Hospital, Kigali, Rwanda; 3 National University of Rwanda, Butare, Rwanda; 4 Gillings School of Global Public Health, University of North Carolina at Chapel Hill, Chapel Hill, NC, USA; 5 Ministry of Health, Republic of Rwanda, Kigali, Rwanda; 6 Department of Obstetrics and Gynecology, University of North Carolina at Chapel Hill, Chapel Hill, NC, USA; 7 Duke Global Health Institute, Duke University, Durham, NC, USA

**Keywords:** Maternal health, Service delivery, Health financing, Health systems, Sub-Saharan Africa

## Abstract

**Background:**

The proportion of births attended by skilled health personnel is one of two indicators used to measure progress towards Millennium Development Goal 5, which aims for a 75% reduction in global maternal mortality ratios by 2015. Rwanda has one of the highest maternal mortality ratios in the world, estimated between 249–584 maternal deaths per 100,000 live births. The objectives of this study were to quantify secular trends in health facility delivery and to identify factors that affect the uptake of intrapartum healthcare services among women living in rural villages in Bugesera District, Eastern Province, Rwanda.

**Methods:**

Using census data and probability proportional to size cluster sampling methodology, 30 villages were selected for community-based, cross-sectional surveys of women aged 18–50 who had given birth in the previous three years. Complete obstetric histories and detailed demographic data were elicited from respondents using iPad technology. Geospatial coordinates were used to calculate the path distances between each village and its designated health center and district hospital. Bivariate and multivariate logistic regressions were used to identify factors associated with delivery in health facilities.

**Results:**

Analysis of 3106 lifetime deliveries from 859 respondents shows a sharp increase in the percentage of health facility deliveries in recent years. Delivering a penultimate baby at a health facility (OR = 4.681 [3.204 - 6.839]), possessing health insurance (OR = 3.812 [1.795 - 8.097]), managing household finances (OR = 1.897 [1.046 - 3.439]), attending more antenatal care visits (OR = 1.567 [1.163 - 2.112]), delivering more recently (OR = 1.438 [1.120 - 1.847] annually), and living closer to a health center (OR = 0.909 [0.846 - 0.976] per km) were independently associated with facility delivery.

**Conclusions:**

The strongest correlates of facility-based delivery in Bugesera District include previous delivery at a health facility, possession of health insurance, greater financial autonomy, more recent interactions with the health system, and proximity to a health center. Recent structural interventions in Rwanda, including the rapid scale-up of community-financed health insurance, likely contributed to the dramatic improvement in the health facility delivery rate observed in our study.

## Background

Maternal mortality remains a pressing problem in the developing world. To date, few countries are on track to meet Millennium Development Goal 5 (MDG 5), which aims for a 75% reduction in maternal mortality by 2015 [1,2]. The statistics are particularly alarming in sub-Saharan Africa, where the lifetime risk of dying from childbirth is 1 in 31, as compared to 1 in 4,300 in industrialized countries [3]. Rwanda, a densely populated country of 11 million people located in central Africa, has made impressive strides in improving its health indicators since the 1994 genocide, but still has one of the highest maternal mortality ratios in the world, estimated between 249–584 maternal deaths per 100,000 live births [1].

The proportion of births attended by skilled health personnel is one of two indicators used to track progress toward achieving MDG 5 [4]. While the debate over whether skilled attendance truly results in decreased maternal mortality remains unsettled, skilled attendance has nevertheless been adopted as a proxy indicator for maternal deaths in the developing world [5-8]. Moreover, widespread reliance on this indicator has contributed to a major shift in global policies aimed at reducing rates of maternal mortality: rather than training and equipping traditional birth attendants, efforts have increasingly been directed at improving access to and utilization of formal healthcare services during the antenatal period and at the time of delivery [5,7].

Of the eight MDG regions, sub-Saharan Africa has shown the slowest progress in increasing the proportion of births attended by skilled health personnel [6]. In Rwanda, although significant progress has been made in increasing the rates of utilization of antenatal care (ANC), improvements in the rates of utilization of delivery care have lagged. The 2007–2008 Interim Demographic and Health Survey (DHS) indicated that 95.8% of Rwandan women attended at least one ANC visit, but that only 45.2% delivered at health facilities [9]. While the 2010 DHS shows improvement in virtually all maternal health indicators, highlighted by the 98.0% of women who reported receiving some ANC, still only 68.9% of Rwandan women reported delivering in a health facility in the five years preceding data collection [10].

Although the overall trends in these Rwanda DHS data are encouraging, the data lack granularity and provide little insight into the factors that impact women’s choice of delivery location. While studies investigating this research question have been conducted in East Asia, the Indian subcontinent, and elsewhere in sub-Saharan Africa, including in neighboring Uganda, Kenya, and Tanzania [8,11-19], there is a clear need for country-specific data given the culturally and locally specific findings of previous studies, in addition to the dramatic changes that have been made to the structure and financing of the Rwandan healthcare system in recent years [20]. The objectives of this study were to quantify secular trends in health facility delivery and to identify factors that affect the uptake of intrapartum healthcare services among women living in rural villages in Bugesera District, Eastern Province, Rwanda.

## Methods

### Study site

**Figure 1:**
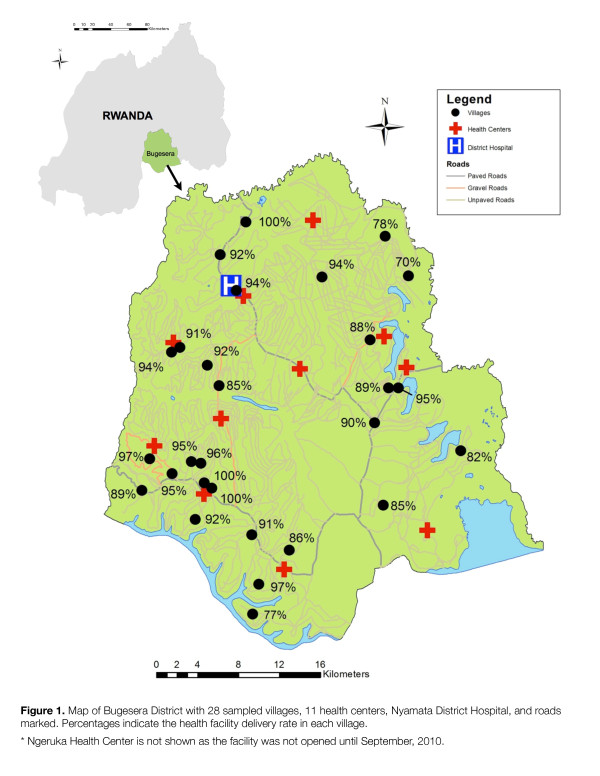
**Map of Bugesera District with 28 sampled villages, 11 health centers, Nyamata District Hospital, and roads marked. Percentages indicate the health facility delivery rate in each village.** * Ngeruka Health Center is not shown as the facility was not opened until September, 2010.

Bugesera, one of seven districts in the Eastern Province of Rwanda, comprises an area of 1337 km^2^ and borders Burundi on its southern edge (see Figure 1) [21]. The district is divided into 15 sectors (*umurenge*), which are in turn divided into 72 cells (*akagari*) and sub-divided into 580 villages (*umudugudu*). According to the most recent district census, Bugesera has a population of 299,630 people, 53.2% of which is female [22]. It is a primarily rural district with one main town, Nyamata, serving as the district capital.

All but three of Bugesera’s 15 sectors have health centers, which function as primary health facilities and are staffed exclusively by nurses. In addition to the 12 health centers, there is a district hospital in Nyamata that employs 12 medical doctors. Nyamata District Hospital is the only facility in the district that offers blood transfusions, operative deliveries, and Cesarean sections. There has been the recent addition of health posts in this district, but these are primarily for the provision of outreach activities and not obstetric deliveries. If needed, patients are transported to Kigali University Teaching Hospital or King Faisal Hospital (both located in the capital, Kigali), which serve as the tertiary referral hospitals for the district.

According to the Rwandan Ministry of Health, the child mortality rate in Bugesera is 72.0 per 1,000 live births (national average = 42.0 per 1,000 live births, rank 29 of 30 districts), while the infant mortality rate is 68.0 per 1,000 live births (national average = 60.5 per 1,000 live births, rank 21 of 30 districts). Unfortunately, district level maternal mortality rates are not available secondary to small sample size. The Ministry’s data further suggest that 66.1% of women in Bugesera deliver in health facilities (national average = 69.2%, rank 16 of 30 districts), 97.4% attend at least one ANC visit (national average = 98.0%, rank 20 of 30 districts), and 21% attend at least four visits (national average = 35.4%, rank 29 of 30 districts) [23]. Thus, on the whole, Bugesera has more marginal health indicators as compared to other districts in Rwanda.

### Study design and survey instrument

A cross-sectional community-based study was conducted in Bugesera District during a six-week period in March and April, 2011. A structured, quantitative survey was used to interview clusters of women aged 18–50 who had given birth in the three years preceding data collection.

The survey instrument was developed following a comprehensive literature search of quantitative and qualitative studies investigating similar research questions in low- and middle-income countries. In addition to demographic and household asset questions, respondents were asked to provide the year, delivery location, and outcome (under-1 death, under-5 death, childhood/adult death, offspring alive) for every pregnancy that progressed past 28 weeks. Data collectors obtained an abbreviated medical and surgical history followed by a complete obstetric history using simplified questions aimed at eliciting any history of prenatal, intrapartum, or postpartum complications. Respondents were then asked more specific questions about their last delivery, including questions regarding ANC utilization, interactions with community health workers, and details of the delivery itself.

The survey was translated into French and Kinyarwanda and back-translated into English to ensure fidelity. After ethical approval was obtained from the Kigali University Teaching Hospital Ethics Committee and the Duke University Health System Institutional Review Board, the survey instrument was converted to an electronic format using FileMaker Pro 11.0v2 (FileMaker Inc., Santa Clara, CA, USA) and uploaded to 3G iPads (Apple Inc., Cupertino, CA, USA) equipped with FileMaker Go 1.1 (FileMaker Inc., Santa Clara, CA, USA). An HTML script was written into the FileMaker Pro database in order to collect geospatial coordinates for each household using the iPads’ 3G connection.

Following a week-long training period, six native Kinyarwanda speakers piloted the survey in non-sampled villages in Bugesera District to test for clarity and functionality. Based on feedback from respondents and data collectors, slight modifications were made to survey questions and FileMaker Pro programming.

### Sampling and sample size

From a sampling frame of 580 villages in Bugesera District, 30 villages were randomly selected using the number of households from the 2002 census and probability proportional to size sampling methodology [24]. Data collectors were instructed to start at the main road at the edge of each village, approach the closest household, determine whether there was a woman within the household who met inclusion criteria (age 18–50 and obstetric delivery within previous three years), and obtain verbal informed consent. Data collectors systematically proceeded to the next household until they had either (a) approached every household, or (b) interviewed a total of 40 women in the village. With census data indicating a range of 26 to 306 households in sampled villages, an average household size of 4.5, and an estimated birth rate of 41 per 1,000 women, the cutoff of 40 women per village was chosen such that all eligible women would be enrolled from most villages while ensuring that the sample would not be dominated by a few large villages.

### Data analysis

Completed survey instruments were downloaded from each iPad and exported from FileMaker Pro to Microsoft Excel (Microsoft Corporation, Redmond, WA, USA) on a nightly basis. Regular data quality assurance activities were conducted and faulty survey instruments were excluded from the final database. Statistical analyses were conducted using Stata 11.2 (StataCorp, College Station, TX, USA). The mode of the household geospatial coordinates in each village was used to describe the location of each study village. Using shape files obtained from Rwanda’s National Institute of Statistics, a distance analysis was run in ArcGIS 10.0 (Esri, Redlands, CA, USA) to calculate the distances traveled along roads between each village and its designated health center and Nyamata District Hospital.

Secular trends in delivery locations were described using data on all deliveries reported by respondents. Using only data from each participant’s last delivery, bivariate and multivariate logistic regression were used to describe associations between delivery location and a variety of covariates. Models were estimated with robust standard errors to account for clustering at the village level.

## Results

**Figure 2:**
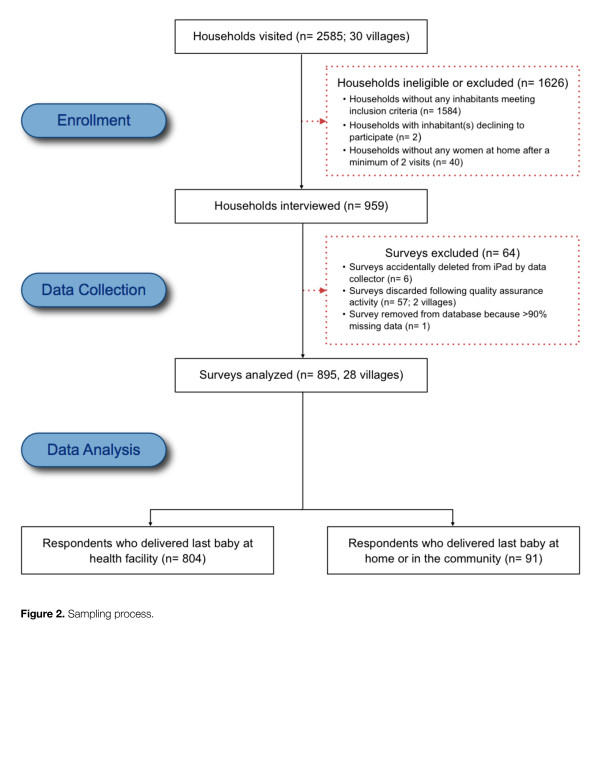
Sampling process.

A total of 959 women meeting inclusion criteria were interviewed, of which 895 were retained in the final database (see Figure 2). The ages of the respondents ranged from 18–49 with a mean age of 29.4 years (SD 6.3). Over half (54.4%) of the respondents were married and an additional 31.2% were living with a man. Nearly 20% had never attended school, while the mean number of years of schooling among those who attended was 5.1 (SD 2.3). The primary occupation of the sampled women was subsistence agriculture, accounting for 90.1% of respondents. More than 86% of respondents were subscribers of a national community-funded health insurance program known as *Mutuelle de Santé*, while 10.9% did not have any form of health insurance. The majority of respondents (57.4%) had given birth between 2–5 times, with a mean parity of 3.5 (SD 2.3) (see Table 1).

**Table 1:** General characteristics of 895 sampled women from 28 villages in Bugesera District, Rwanda

	**N**	**%**
Age		
18-24	209	23.4%
18-24	209	23.4%
25-33	468	52.3%
34-49	217	24.2%
No response	1	0.1%
Marital Status		
Married	487	54.4%
Living together	279	31.2%
Single (never married)	73	8.2%
Separated/divorced	44	4.9%
Widowed	12	1.3%
Education		
No education	178	19.9%
Primary	636	71.1%
Secondary or higher	81	9.1%
Religion		
Protestant	374	41.8%
Catholic	310	34.7%
Adventist	204	22.8%
Muslim	6	0.7%
No response	1	0.1%
Profession		
Agriculture	806	90.1%
Self-employed/small business owner	38	4.2%
No work outside the home	24	2.7%
Unskilled employee	22	2.5%
Student	5	0.6%
Insurance		
* Mutuelle de Santé*	772	86.3%
State Insurance (RAMA)	14	1.6%
Military Insurance (MMI)	9	1.0%
Private	2	0.2%
None	98	10.9%
Parity		
1	208	23.2%
2-5	514	57.4%
6-13	173	19.3%

### Utilization of antenatal care

Almost 99% of respondents reported that they received some ANC during their last pregnancy. Over 95% attended at least two visits and 80.0% attended at least three visits, but only 31.7% attended four or more visits as recommended by the Rwandan Ministry of Health. The mean timing of the first ANC visit was at 4.1 months of pregnancy (SD 1.4), with just 40.8% of women attending their first ANC visit during the first trimester. Of women who attended at least one ANC visit, 86.6% were accompanied by their husband or partner on one or more occasion. Further, of the respondents who attended ANC, 94.4% reported that they were advised by a doctor, nurse, or TBA to deliver at a health facility. Forty-three percent of respondents stated that they were visited by a community health worker (CHW) during their pregnancy and 95.9% of these reported that they were advised by a CHW to deliver at a health facility.

### Utilization of delivery care

During their most recent delivery, 89.8% of respondents reported delivering in a health facility. Of these, 80.2% delivered in a health center, 16.8% delivered at a district hospital, and 3.0% delivered in a hospital in Kigali. Overall, 88.7% of women reported that a doctor and/or nurse attended their delivery. Among women who delivered in a health facility, 98.5% benefitted from skilled attendance, compared to only 2.2% of women who delivered in the community. According to respondent recall, 89.1% of all deliveries were vaginal, 7.7% were Cesarean sections, and 3.2% were operative (vacuum or forceps).

Among respondents who delivered at a health facility, it took a mean of 52.7 minutes (SD 36.1) to reach the delivery location and the mean cost of transportation was 0.97 USD (SD 2.6). The median distance along roads from sampled villages to their designated health centers was 5.8 km (SD 3.9, min = 0.9 km, max = 13.7 km), while the median distance to Nyamata District Hospital was 24.8 km (SD 10.4, min = 1.3 km, max = 13.7 km).

**Figure 3:**
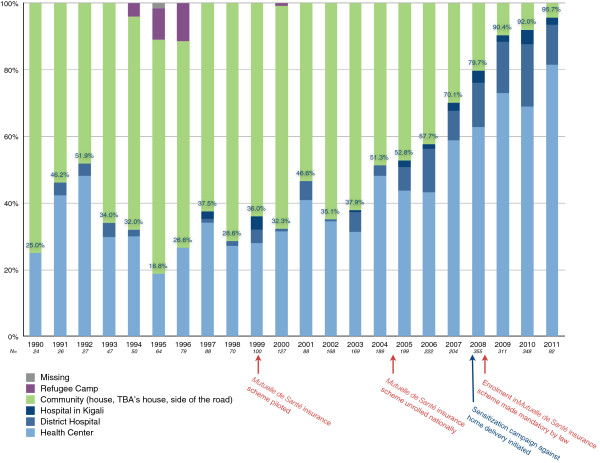
Breakdown of delivery locations for lifetime deliveries of 895 sampled women over time.

Figure 3 plots the location of all deliveries since 1990 over time. The data used to generate this figure was derived from a series of questions that asked respondents to list the year and delivery location for every pregnancy that progressed past 28 weeks, as described in the methods section. These data show an increase in health facility delivery rates in the sampled population over time, with a notable surge between 2006–2007 (from 57.7% to 70.1%), and another between 2008–2009 (from 79.7% to 90.4%).

### Determinants of utilization of delivery care

The results of both the bivariate and multivariate logistic regressions are provided in Table 2. In the bivariate analysis, health facility delivery was more common among younger women (OR = 0.908 [0.874 - 0.943] per year of age) and decreased with increasing parity (OR = 0.795 [0.738 - 0.856] for each additional birth). Women who had more schooling were more likely to deliver in a health facility (OR = 1.136 [1.042 - 1.239] per year of schooling). The education levels of the respondents’ mothers, however, were not significant.

**Table 2:** Bivariate and multivariate logistic regression analysis correlates of health facility delivery for last deliveries among 895 women in Bugesera District, Rwanda

	**Bivariate**	**Multivariate**
	**(N=891-895)**^**$**^	**(N=882)**^**$**^
Age^‡^	0.908***	0.941
(18–49 years)	[0.874 - 0.943]	[0.872 - 1.015]
Married	1.188	0.783
(unmarried vs. married)	[0.662 - 2.135]	[0.430 - 1.424]
Respondent’s education (years of education)^‡^	1.136**	1.052
(0–13)	[1.042 - 1.239]	[0.975 - 1.135]
Mother's education	1.319	0.909
(no education vs. any education)	[0.774 - 2.249]	[0.524 - 1.580]
Financial control	1.690*	1.897*
(no control over family finances vs. some control)	[1.063 - 2.687]	[1.046 - 3.439]
Health insurance	5.683***	3.812***
(no health insurance vs. health insurance)	[3.186 - 10.14]	[1.795 - 8.097]
Parity^‡^	0.795***	0.873
(1–13)	[0.738 - 0.856]	[0.726 - 1.049]
Next-to-last delivery at facility	3.660***	4.681***
(no vs. yes)	[2.394 - 5.596]	[3.204 - 6.839]
Any offspring death	0.468**	0.84
(no vs. yes)	[0.293 - 0.748]	[0.406 - 1.741]
Number of past intra/postpartum complications^‡^	1.273	1
(0–4)	[0.808 - 2.006]	[0.582 - 1.719]
Any prenatal complications	1.309	1.122
(no vs. yes)	[0.653 - 2.625]	[0.527 - 2.386]
Number of antenatal clinic visits^‡^	1.899***	1.567**
(0–9)	[1.473 - 2.447]	[1.163 - 2.112]
Any antenatal clinic visits with partner	1.949**	1.246
(no vs. yes)	[1.235 - 3.075]	[0.726 - 2.141]
Community health worker visits^‡^	1.069	1.173
(0–20)	[0.925 - 1.236]	[0.954 - 1.442]
Use of non-obstetric services at health facility	1.738*	1.171
(no vs. yes)	[1.091 - 2.768]	[0.704 - 1.950]
Advised by healthcare provider	1.785	1.619
(no vs. yes)	[0.787 - 4.051]	[0.534 - 4.908]
Timing of labor	1.089	1.076
(night vs. day)	[0.665 - 1.783]	[0.608 - 1.907]
Distance (kilometers)^‡^	0.888***	0.909**
(0–14)	[0.839 - 0.941]	[0.846 - 0.976]
Time (year)^‡^	1.578***	1.438**
(2008–2011)	[1.254 - 1.986]	[1.120 - 1.847]

Odds ratios and 95% confidence intervals; standard errors adjusted for clustering at the village level.

*denotes p<0.05, **denotes p<0.01 ***denotes p<0.001 ‡ denotes a continous variable

Participation in the management of household finances (OR = 1.690 [1.063 - 2.687]) was predictive of health facility delivery, as was possessing health insurance (OR = 5.683 [3.186 - 10.14]). Greater distance between the respondent’s village and her designated health center was negatively associated with facility delivery (OR = 0.888 [0.839 - 0.941] per additional kilometer), while the timing of labor commencement (day versus night) was not predictive of delivery location. A history of an offspring death was associated with decreased health facility delivery (OR = 0.468 [0.293 - 0.748]). A history of prenatal, intrapartum, or postpartum complications, on the other hand, did not predict increased facility delivery.

Utilization of non-obstetric healthcare services (OR = 1.738 [1.091 - 2.768]) and delivering a penultimate baby at a health facility (OR = 3.660 [2.394 - 5.596]) were associated with higher rates of facility delivery, as were an increasing number of ANC visits (OR = 1.899 [1.473 - 2.447]) and attending an ANC visit with one’s partner (OR = 1.949 [1.235 - 3.075]). The number of visits by a CHW was not significant, nor was being advised by a healthcare provider to deliver at a health facility. Lastly, more recent delivery year (2008 to 2011) was associated with increased facility delivery (OR = 1.578 [1.254 - 1.986] per year).

In a multivariate logistic regression analysis, delivering a penultimate baby at a health facility (OR = 4.681 [3.204 - 6.839]), possessing health insurance (OR = 3.812 [1.795 - 8.097]), participating in the management of household finances (OR = 1.897 [1.046 - 3.439]), attending a greater number of ANC visits (OR = 1.567 [1.163 - 2.112]), delivering more recently (OR = 1.438 [1.120 - 1.847] per year), and living in a village located closer to a health center (OR = 0.909 [0.846 - 0.976] per kilometer) were independently associated with facility delivery. Age and parity were jointly significant (not shown); however, due to their high correlation (rho = 0.470) it was not possible to disentangle their independent effects in the multivariate model. The Hosmer-Lemeshow goodness of fit test suggests no problems concerning the fit of our multivariate model (p = 0.571) [25].

## Discussion

Our study had two major findings. First, there has been a remarkable increase in the rate of health facility delivery in Bugesera District over time, with a particularly sharp increase over the past five years. Since 2009, over 90% of sampled women in Bugesera District delivered in health facilities; in contrast, only 42.9% of all sub-Saharan African women deliver in health facilities [8]. Second, multivariate logistic regression analysis suggests that delivering a penultimate baby at a health facility, possessing health insurance, having some control over household finances, attending more ANC visits, delivering more recently, and living closer to health centers are significantly associated with facility delivery.

The Government of Rwanda has set a progressive agenda towards improving its health indicators since the 1994 genocide, which left approximately one million people dead and the country’s health infrastructure destroyed. Given the multitude of initiatives that have been introduced over the past 10–15 years, it is difficult to ascertain which structural intervention contributed most significantly to the improved uptake of skilled delivery care shown in our study. In reality, a number of interventions likely played a role, including the commitment of a dedicated political leadership to improved donor aid coordination; the refurbishment of most health facilities and many roads in the country; the implementation of a nation-wide performance-based payment initiative for healthcare providers; the national rollout of a community-financed health insurance program known as *Mutuelle de Santé*; the steady rise in overall health insurance subscription rates, from 68% household coverage in the 2007–8 Interim DHS to 78% household coverage in the 2010 DHS; and the inauguration of an aggressive sensitization campaign linked to performance contracts at the district level (*imihigo*) that aims to educate women on the dangers of delivering at home while strongly encouraging health facility delivery [9,10,20,26-28]. Disentangling the potential effects of each of these reforms in Rwanda’s health sector is impossible. It is noteworthy, however that at least one detailed analysis of Ghana’s National Health Insurance Scheme concluded that its implementation was not associated with increased use of maternal health services. Rather, the poor quality of facilities, cultural preferences to deliver at home, and lack of transportation were considered significant barriers [29].

While it is not clear from our data whether the observed increase in facility-based delivery in Bugesera District resulted in decreased maternal mortality, our results are in and of themselves encouraging. Given the impressive percentage of women presenting for delivery at health facilities, a reasonable next step in addressing maternal mortality in Bugesera District would be to shift focus from promoting skilled attendance to improving the quality of ANC and delivery care at health facilities, primarily by upgrading the knowledge of health professionals so as to meet WHO standards for skilled attendants [4]. This is especially important given the demonstrated lack of competence among purportedly skilled attendants in Rwanda and other developing countries when judged against WHO standards [30-32].

There are several possible limitations to our study. First, as in all cross-sectional studies, we can infer association but not causation from our results. Second, social desirability bias could have artificially inflated the reported rates of health facility delivery; however, the study was confidential and data collectors were instructed to assure women that their responses could not be linked to them. Third, our sample did not include adolescents younger than 18 years old. Regression analysis indicates that younger women were more likely to deliver in health facilities, suggesting that inclusion of this group may have led to increased overall facility delivery rates. Finally, our study was limited to 28 villages in one district and thus the findings cannot be extrapolated to the country as a whole; on the other hand, trends in facility-based deliveries between 2006–2010 in our data were similar to trends observed in the most recent DHS [10]. While the health facility delivery rate for our sample during this time period (80.2%) was higher than the country-wide rate (68.9%) reported by the 2010 DHS, national data show similar trends, suggesting that our district-wide sample was not dissimilar to the latest DHS sample. For this reason, it is reasonable to assume that factors that correlate with delivery uptake in health facilities in Bugesera District would likely play a similar role in other districts in Rwanda. Moreover, even though it is not possible to generalize our results to the country as a whole, having district-level data is particularly important in Rwanda given the recent implementation of a comprehensive national decentralization strategy. Among other changes, decentralization yielded 30 districts, which function as the unit of implementation for major central government policies and social programs; accordingly, having estimates of maternal health indicators at the district level will inform planning, budgeting, and monitoring processes.

## Conclusions

The dramatic improvement in the health facility delivery rate in Bugesera District likely reflects a number of important structural interventions, including the rapid scale-up of the *Mutuelle de Santé*, to which a considerable majority of Rwandans now subscribe. If the trend in improved uptake of skilled delivery care in Bugesera holds true for the nation, Rwanda should make substantial progress towards achieving MDG 5. While further research is needed to determine how best to encourage facility-based delivery among those women who continue to deliver in the community, the results of our multivariate analysis lead us to suggest that sensitization campaigns target women who delivered their last baby at home and/or attended few ANC visits. Further, we argue that renewing efforts to achieve universal health insurance and to promote women’s empowerment, in addition to increasing access to transportation services, could also help to promote facility deliveries in the district.

## Abbreviations

ANC: Antenatal Care; CHW: Community Health Worker; DHS: Demographic Health Survey; MDG: Millennium Development Goal.

## Competing interests

The authors declare that they have no disclosures or competing interests.

## Authors’ contributions

SJ, NT, JW, JO, and SR designed the study. SJ, FN, and AW executed the study. SJ, JO, NT, and AW completed the analysis. SJ authored the manuscript and received additional review and input from NT, JW, JO, AW, and FS. All authors read and approved the final manuscript.

## Pre-publication history

The pre-publication history for this paper can be accessed here:

http://www.biomedcentral.com/1471-2458/12/1049/prepub
